# Spinacetin, an Anti-Inflammatory Natural Compound with Multiple Pharmacological Properties

**DOI:** 10.3390/cimb48030250

**Published:** 2026-02-26

**Authors:** Varun Jaiswal, Hae-Jeung Lee

**Affiliations:** 1 Department of Food and Nutrition, College of BioNano Technology, Gachon University, 1342 Seongnam-daero, Sujeong-gu, Seongnam-si 13120, Gyeonggi, Republic of Korea; computationalvarun@gmail.com; 2 Institute for Aging and Clinical Nutrition Research, Gachon University, Seongnam-si 13120, Republic of Korea; 3 Department of Health Sciences and Technology, Gachon Advanced Institute for Health Sciences and Technology (GAIHST), Gachon University, Incheon 21999, Republic of Korea

**Keywords:** spinacetin, flavonol, pharmacological properties, anti-inflammatory, antioxidant, molecular mechanism, molecular targets, pathways, docking

## Abstract

Spinacetin, a flavonol initially isolated from *Spinacia oleracea*, is a key bioactive constituent in various plants. In previous studies, spinacetin has shown various potential pharmacological properties, including anti-inflammatory, antioxidant, anticancer, antidiabetic, antileishmanial, analgesic, muscle relaxant, sedative, spasmolytic, anti-nephrolithiatic, and depigmentation. It has also shown a protective effect on the vital organs, such as the liver and heart. The widely reported antioxidant and anti-inflammation activities of spinacetin may contribute to its multiple pharmacological properties. In silico molecular docking studies also supported the important activities of spinacetin, such as anticancer, antidiabetic, and hepatoprotective activities. In molecular studies, spinacetin was found to act on targets such as α-glucosidase, Bax, Bcl-2, COX-2, CAT, GPx, and SOD and modulates crucial signaling pathways, including SIRT1/AMPK/mTOR, AMPK/SIRT1/PGC-1α, MAPK, NF-κB, and AKT/IkBα/NF-kB pathways. These target pathways are associated with numerous important diseases and conditions, which highlights the high pharmacological potential of spinacetin. Still, most of the pharmacological activities of spinacetin are in initial preclinical phases. Limitations of pharmacokinetics and safety studies also restrict its development. By addressing the current limitations and lack of integrated data on spinacetin’s pharmacological properties, molecular mechanisms, and pathways, this work provides a foundational resource for its future clinical and therapeutic development.

## 1. Introduction

Flavonols are an important class of flavonoids found in many vegetables and fruits, and they are known for their diverse biological activities [1]. Spinacetin (3,5,7-trihydroxy-2-(4-hydroxy-3-methoxyphenyl)-6-methoxychromen-4-one) is a flavonol that was first isolated from the leaves of spinach (*Spinacia oleracea*), from which it got its name [2]. However, spinacetin has been identified in numerous plant species, including several species used in traditional medicine, such as *Artemisia copa*, *Euphorbia pulcherrima*, *Chrysanthemum morifolium*, as well as various species within the *Inula* and *Pistacia* genera. These plants are known for their multiple pharmacological properties, including antioxidant, anti-inflammatory, and anticancer activities [3,4,5,6]. Supported by in vitro, in vivo, and in silico studies, spinacetin is considered one of the main contributors to the diverse medicinal properties of these plants. Therefore, various pharmacological activities of spinacetin were evaluated after its isolation from these plants.

In recent studies, spinacetin has shown pharmacological potential against serious diseases and conditions, such as cancer, diabetes, and liver and cardiotoxicities [7,8,9,10]. Potential analgesic, muscle-relaxant, sedative, and spasmolytic effects of spinacetin were also observed in the animal studies [11]. Preliminary in vitro studies against enzymes such as urease, tyrosinase, and phosphodiesterase 1 have shown its potential against kidney stones, hyperpigmentation, and neurological disorders (such as Alzheimer disease) [12,13,14]. Spinacetin recently showed potent antileishmanial activity, highlighting its potential in treating infectious diseases. Spinacetin’s diverse pharmacological activities are primarily driven by its potent anti-inflammatory and antioxidant effects. These properties have been validated through various in vitro, in vivo, and in silico studies [9,15]. After experimental studies, in silico molecular docking analysis also supported the different pharmacological activities, such as anticancer, antidiabetic, anti-inflammatory, and hepatoprotective activities [8,9].

In some pharmacological studies, spinacetin exhibited greater potency than the positive controls used in the respective experiments, including 7-deazaxanthine and baicalein (anticancer), as well as loratadine and ibuprofen (anti-inflammatory). The effective pharmacological profile of spinacetin has inspired researchers to decipher its molecular mechanisms. These studies revealed that spinacetin along with marker genes acts on key targets such as B-cell lymphoma 2 (Bcl-2), Bcl-2 associated X protein (Bax), cyclooxygenase-2 (COX-2), catalase (CAT), glutathione Peroxidase (GPx), inducible nitric oxide synthase (iNOS) and superoxide dismutase (SOD), and also modulating crucial signaling pathways including Sirtuin 1(SIRT1)/5′ adenosine monophosphate-activated protein kinase (AMPK)/Mechanistic Target of Rapamycin (mTOR), AMPK/SIRT1/Peroxisome proliferator-activated receptor gamma coactivator 1(PGC-1)-α, Mitogen-Activated Protein Kinase (MAPK), Nuclear Factor kappa-light-chain-enhancer of activated B cells (NF-κB), and AKT/NF-κB cells inhibitor-α (IkBα)/NF-kB pathways. These targets are crucial in several important diseases, which justify multiple pharmacological properties of spinacetin and highlight its high potential for development as a therapeutic agent [16].

In spite of several pharmacological properties and potential against serious diseases and conditions, pharmacological development of spinacetin is still in an early phase (preclinical stage). Limitation of pharmacokinetic studies of spinacetin also restricts its pharmacological development. However, after bolus ingestion of intrinsically labeled spinach with ^13^CO_2_, the spinacetin was detected in the human plasma [17]. A comprehensive compilation of pharmacological properties, mechanisms of action, molecular targets, and associated biological pathways is lacking in the existing literature, which is essential for its therapeutic development against important diseases and conditions. The current work not only provides a compilation of the pharmacological properties, molecular targets, and pathways associated with spinacetin, but also highlights existing gaps and discusses potential future directions that may support its pharmacological development.

## 2. Electronic Literature Search

A structured literature search was conducted using Scopus, PubMed, and Web of Science to identify studies reporting the pharmacological activities of spinacetin. The relevant keywords to study and their combinations, such as “spinacetin”, “spinacetine”, “pharmacological activity”, “anticancer activity”, “anti-diabetic activity”, “anti-inflammatory”, “antioxidant”, “anticancer”, “antidiabetic”, “analgesic”, “muscle relaxant”, “sedative”, “spasmolytic”, “anti-nephrolithiatic”, “depigmentation” were searched on the above mentioned databases. All articles published in English up to December 2025 were considered. The initial database search yielded 101 records from Scopus, 61 from Web of Science, and 34 from PubMed. After removal of duplicate entries, 111 unique records were screened based on title and abstract. Full texts of potentially relevant studies were subsequently evaluated. Studies were included if they reported experimental pharmacological evaluation of isolated spinacetin using in vitro, in vivo, or combined approaches. Studies focusing exclusively on spinacetin derivatives, glycosides, crude extracts without isolation, or solely computational predictions without experimental validation were excluded. Notably, only peer-reviewed articles from the indexed sources within the selected databases were considered in the current work.

## 3. Pharmacological Activities of Spinacetin

The pharmacological importance of spinacetin as a flavonoid [18,19] and its presence in different medicinal plants have led to various studies focused on its activity after isolation. Spinacetin is typically isolated using chromatographic techniques from fractions obtained through sequential solvent extraction of plant material. Considering the importance of natural compounds in drug development [20], researchers have discovered the pharmacological potential of spinacetin against different diseases through various in vitro, in vivo, and in silico methods.

### 3.1. Anti-Inflammatory Activity of Spinacetin

Inflammation is associated with damage to different organs and the pathology of various diseases, including cancer and obesity [21,22,23]. Therefore, the anti-inflammatory activity of spinacetin may support its various pharmacological activities. Significant anti-inflammatory activity of spinacetin has been observed in in vitro and animal studies, which also suggested that it targets key inflammatory pathways.

#### 3.1.1. In Vitro Anti-Inflammatory Activity of Spinacetin

Bioactivity-guided fractionation of the *Artemisia copa* aerial parts extract was performed to identify the key compounds responsible for its anti-inflammatory activity [24]. The powdered, dried aerial parts of the plant were extracted via maceration in dichloromethane at room temperature for 24 h. The resulting marc was dried at room temperature and subsequently re-extracted with ethanol (50%). These extracts were subjected to column chromatography using Sephadex LH-20, yielding 100 fractions. Seven of these fractions were further purified via paper chromatography to yield spinacetin. Researchers identified the isolated compounds using UV spectral analysis, mass spectroscopy (MS) analysis, and comparison with authentic samples. The purity of these compounds was then confirmed using high-performance liquid chromatography (HPLC). Spinacetin, which was obtained in a high yield (3.2 mg) from the corresponding fractions, was selected for anti-inflammatory activity studies alongside other compounds. Inflammatory stimuli, such as lipopolysaccharide (LPS), can induce the expression of COX-2 and iNOS, leading to the overproduction of prostaglandin E2 (PGE_2_) and nitric oxide (NO), respectively. Both mediators are closely associated with the pathogenesis of various inflammatory conditions.

Spinacetin significantly inhibited the production of PGE_2_ and NO, by 31.1% and 37.0%, respectively, in LPS-stimulated RAW 264.7 macrophages. Spinacetin also significantly inhibited the human synovial lipase A_2_ (sPLA_2_) enzyme, which is a key inflammatory mediator found in high concentrations in the synovial fluid of patients with inflammatory arthritis. These results suggest that the anti-inflammatory potential of spinacetin could be extended to the treatment of inflammatory diseases, such as inflammatory arthritis. The study highlighted the potential of flavonoids such as spinacetin present in the extract for anti-inflammatory activity.

Later, the anti-inflammatory activity of spinacetin isolated from *Inula japonica* was evaluated through a series of in vitro and in vivo experiments [25]. Given the role of histamine as a primary mediator in allergic inflammation, the ability of spinacetin to inhibit histamine degranulation was evaluated in IgE/Ag-stimulated bone marrow-derived mast cells (BMMCs). Spinacetin strongly inhibited the release of histamine and reduced intracellular Ca^2+^ levels, both of which were significantly elevated in activated BMMCs. A spleen tyrosine kinase (Syk) inhibitor was employed as a positive control, and the results suggest that spinacetin inhibits mast cell degranulation by suppressing Ca^2+^ mobilization.

Cytosolic phospholipase A_2_ (cPLA_2_) mobilizes arachidonic acid, which generates potent lipid mediators like prostaglandin (PG) D2 and leukotrienes, crucial for inflammation, and allergies [26]. Spinacetin treatment significantly decreased leukotrienes C4 (LTC4) generation, as well as the phosphorylation and nuclear translocation of cPLA_2_, in a dose-dependent manner in IgE/Ag-stimulated BMMCs (Table 1). The enzymatic activity of cPLA_2_ is known to be regulated by MAPK-mediated phosphorylation. Thus, the effect of spinacetin on the phosphorylation of MAPKs was evaluated. Spinacetin significantly attenuated the IgE/Ag-induced phosphorylation of ERK1/2, JNK1/2, and p38. These findings suggest that spinacetin exerts its inhibitory effects on cPLA_2_ activation and subsequent LTC4 synthesis by suppressing the MAPK signaling cascade. Additionally, spinacetin suppressed IL-6 cytokine levels in a dose-dependent manner; this cytokine plays a crucial role in various inflammatory diseases. Furthermore, spinacetin dose-dependently inhibited COX-2 expression, thereby modulating the production of PGs, which are key mediators of the inflammatory response. The pro-inflammatory transcription factor NF-κB positively regulates IL-6 production and COX-2 expression. To validate the involvement of this pathway, the activation of NF-κB was investigated. The activation of NF-κB occurs following the phosphorylation, ubiquitination, and proteasomal degradation of IκBα [27]. Subsequently, the liberated NF-κB translocates from the cytosol into the nucleus to initiate gene transcription. NF-κB translocated from the cytosol to the nucleus in IgE/Ag-stimulated BMMCs; however, spinacetin significantly inhibited this translocation. Furthermore, spinacetin treatment suppressed the phosphorylation and subsequent degradation of IκBα. The Phosphoinositide 3-kinase/Protein Kinase B (PI3K/AKT) pathway is known to activate NF-κB; therefore, the phosphorylation of AKT, a pivotal kinase in this signaling cascade, was also investigated. Western blot analysis revealed that the IgE/Ag-induced increase in AKT phosphorylation was dose-dependently suppressed by spinacetin.

In IgE/Ag-stimulated RBL-2H3 cells, spinacetin exhibited consistent inhibitory effects, including the reduction in cPLA2 phosphorylation and translocation, the attenuation of MAPK signaling (specifically ERK1/2, JNK1/2, and p38 phosphorylation), and the suppression of both AKT phosphorylation and NF-κB activation. Furthermore, spinacetin was found to dose-dependently suppress the phosphorylation of Phospholipase C-gamma (PLCγ), which had been markedly induced by IgE/Ag stimulation (Table 1). Finally, the upstream signaling molecules of IgE-mediated mast cell activation, Syk and linker for activation of T cells (LAT), were examined. The finding that spinacetin suppressed the phosphorylation of both Syk and LAT indicates that the compound inhibits IgE-mediated mast cell activation by modulating Syk-dependent signaling pathways. The potent anti-inflammatory effects observed in cell-based assays and the subsequent suppression of key inflammatory pathways justify further in vivo investigations of spinacetin.

#### 3.1.2. In Vivo Anti-Inflammatory Activity of Spinacetin

In vitro studies have demonstrated that spinacetin possesses potent anti-inflammatory activity in IgE/Ag-stimulated mast cells. Given that mast cells are the primary effector cells in IgE-mediated allergic reactions and anaphylaxis, the anti-allergic effects of spinacetin were further evaluated using a passive cutaneous anaphylaxis (PCA) model in institute of cancer research (ICR) mice, with dexamethasone serving as the positive control [25]. The result showed that the spinacetin significantly suppressed the PCA reaction (by the amount of diffused dye and ear thickness) in a dose-dependent manner. These findings demonstrate that the suppression of mast cell activation observed in vitro serves as the mechanistic basis for the anti-inflammatory activity of spinacetin in the murine PCA mouse model.

Later, Spinacetin was isolated from the chloroform fraction of the methanolic extract of *Euphorbia pulcherrima*, a plant known for its diverse pharmacological activities [4].

The anti-inflammatory activity of spinacetin was evaluated using carrageenan-induced and histamine-induced paw edema models in Balb/c mice. At a dose of 20 mg/kg, spinacetin demonstrated significant anti-inflammatory effects in both models. While its efficacy was lower than the positive control, diclofenac, in the carrageenan model, it surpassed the activity of the control (loratadine) in the histamine-induced model [11]. The high anti-inflammatory activity observed in the histamine-induced model is in line with the in vitro anti-inflammatory activity and mechanism of action, where spinacetin suppressed histamine release from BMMCs [11,25].

The demonstrated anti-inflammatory efficacy of spinacetin across different animal models supports its potential as a therapeutic agent for the treatment of inflammation and inflammation-associated diseases.

#### 3.1.3. In Silico Anti-Inflammatory Activity of Spinacetin

Following the in vivo demonstration of spinacetin’s anti-inflammatory activity, a molecular docking study was performed on the COX-2 enzyme to investigate the potential molecular interactions and underlying mechanisms of action.

In the docking study, spinacetin exhibited effective binding interactions with the COX-2 enzyme, forming hydrogen bonds with His90, Gln192, Ser353, and Arg513. The binding energy and inhibition constant (*Ki*) for this complex were −8.46 kcal/mol and 0.631 μM, respectively. Results from the molecular studies support the in vivo anti-inflammatory activity of spinacetin through inhibition of COX-2 enzyme. Similarly, suppression of COX-2 enzyme in in vitro experiments was also in line with the in silico results.

Finally, it can be concluded that the different animal models (PCA and paw edema models induced by loratadine and histamine) supported by in vitro and in silico experiments revealed the effective anti-inflammatory activities of spinacetin. It clearly showed the potential of spinacetin against other inflammatory diseases and conditions such as inflammatory bowel diseases, rheumatoid arthritis, myocarditis, and psoriasis. The effective suppression of inflammatory activity in IgE/Ag-stimulated mast cells also highlights its potential for allergy and related diseases [28]. Importantly, the anti-inflammatory activity of spinacetin can be compared with structurally similar and extensively studied phytocompounds, such as luteolin, kaempferol, and quercetin. Like spinacetin, these compounds also target the NF-κB and MAPK signaling pathways to exert their anti-inflammatory effects [29,30,31]. However, while clinical studies have supported the anti-inflammatory potential of these flavonoids in inflammation-associated diseases, such studies have not yet been conducted for spinacetin [29,30].

### 3.2. Anti-Cancer Activity of Spinacetin

The anti-inflammatory activity of spinacetin has been demonstrated across various in vitro and in vivo models. This bioactivity is particularly significant given that anti-inflammation is a primary mechanism driving its broader anticancer potential. The anticancer activity of spinacetin, isolated from medicinal plants, was evaluated through various in vitro and in silico experiments against multiple cancer types and multidrug-resistant (MDR) cell lines.

#### 3.2.1. In Vitro Anticancer Activity of Spinacetin

A study was conducted to identify the bioactive compounds responsible for the known anticancer activity of *Pistacia integerrima* extract [10]. Spinacetin and five other flavonoids isolated from the extract of *Pistacia integerrima* were screened for their anticancer potential against different cancer cell lines, including HepG2 (hepatoma G2), A498 (kidney cell lines), NCI-H226 (lung carcinoma), and MDR2780 AD (MDR human ovarian carcinoma cell line) [10]. Across most cell lines tested, 3-(4,5-dimethylthiazole-2-yl)-2,5-diphenyl tetrazolium bromide (MTT) assay revealed that spinacetin was the second most potent compound, following patuletin, in terms of growth inhibition. The effective inhibitory activity against the MDR human ovarian carcinoma cell line highlights the potential of spinacetin in treating drug-resistant ovarian cancer (Table 2). The anticancer properties of the extract of *Pistacia integerrima* appear to stem from its constituent flavonoids; these interactions were subsequently characterized using in silico docking studies to identify potential binding mechanisms.

In another study, spinacetin isolated from the galls of *Pistacia chinensis* was investigated for its anticancer activity through the inhibition of aldose reductase and thymidine phosphorylase enzymes in in vitro experiments, along with other compounds [32]. The pharmacological inhibition of aldose reductase is emerging as a pivotal strategy in medicinal chemistry to counteract metabolic reprogramming in malignancies [33]. Similarly, thymidine phosphorylase is a potential anticancer target owing to its role in angiogenesis and tumor growth [34]. Spinacetin showed the highest inhibitory activity against both aldose reductase and thymidine phosphorylase compared to the other tested compounds. In the case of thymidine phosphorylase, spinacetin exhibited even higher activity than the positive control, 7-deazaxanthine, used in the inhibition experiment. The results supported competitive and noncompetitive mechanisms of aldose reductase and thymidine phosphorylase inhibition by spinacetin [32].

In a recent study, researchers isolated three main compounds (spinacetin, patuletin, and pistagremic acid) of *Pistacia integerrima* to analyze their inhibitory effects on lipoxygenase [8]. Lipoxygenase serves as a key therapeutic target in both cancer and inflammation, two processes that are pathophysiologically intertwined. In the lipoxygenase inhibition assay, spinacetin was found to be the most active among the isolated compounds, even outperforming baicalein, one of the two positive controls used in the study [8]. However, the MTT assay revealed no cytotoxicity of spinacetin toward human fibroblast cells, suggesting that the concentrations used in this study were safe. Furthermore, in silico docking was employed to identify potential molecular targets of spinacetin and characterize its binding interactions with the specific proteins evaluated in the in vitro anticancer assays.

#### 3.2.2. In Silico Anticancer Activity

Following the demonstration of the anticancer activity of spinacetin and other flavonoids against a panel of cancer cell lines, including an MDR cells, molecular docking simulations were performed to evaluate their affinity for the colchicine-binding site of tubulin [10]. Method validation was performed by redocking the co-crystal ligand (colchicine) back into the protein structure to verify the reproducibility of the docking protocol. In the docking study, spinacetin exhibited efficient binding affinity through various interactions. While spinacetin may exhibit anticancer effects through multiple molecular targets, the current investigation was limited to its interaction with the colchicine-binding site of tubulin; other potential targets remain beyond the scope of this study.

Docking studies on two other important targets, aldose reductase and thymidine phosphorylase, were also conducted after spinacetin demonstrated effective inhibition in in vitro experiments [32]. The crystal structures of both enzymes were retrieved from the Protein Data Bank (PDB), and redocking of the cognate ligands was performed to validate the docking protocol. The results showed that spinacetin interacted with reported catalytic residues, in the case of aldose reductase. Similarly, interaction analysis revealed that spinacetin binds to the nucleoside-binding residues of thymidine phosphorylase, which are essential for its catalytic machinery. Consistent with the in vitro findings, spinacetin exhibited the highest binding affinity for this enzyme, surpassing the docking score of the positive control (the cognate ligand).

In a recent study, after observing the effective inhibition of the lipoxygenase enzyme by spinacetin, a molecular docking study was conducted to validate and decipher the binding of spinacetin to the enzyme [8]. Validation of the docking protocol was confirmed by redocking the co-crystallized ligand, which yielded a low root mean square deviation (RMSD) value (0.09 Å). Spinacetin exhibited a docking score of −6.074 kcal/mol, and interaction analysis of the docked complex revealed that the compound forms a robust interaction with the lipoxygenase enzyme. Together, effective inhibition of enzymes in vitro assays, strong interaction profile observed in the docking study and no toxicity on normal human cells suggested the anticancer potential of spinacetin in the study. Overall, inhibition of different types of cancer cells (liver, kidney, lung, and ovarian) by spinacetin and in vitro suppression of general cancer targets (lipoxygenase, aldose reductase, and thymidine phosphorylase) directly supported by in silico docking studies suggested the anticancer potential against multiple cancer types. However, the anticancer potential of spinacetin has not yet been evaluated in animal models. Such studies are highly recommended, as pharmacokinetics and bioavailability significantly influence in vivo anticancer efficacy. Thus, animal studies are crucial to establish its therapeutic potential.

Compared to spinacetin, similar phytocompounds such as kaempferol and quercetin have been extensively studied across a broader range of cancer types. Numerous in vivo studies have validated the anticancer effects of these comparable flavonoids, with mechanistic investigations demonstrating their ability to target diverse oncogenic signaling pathways [29,35]. Consequently, it would be valuable for future studies to simultaneously evaluate the anticancer activity of spinacetin alongside these phytocompounds, exploring whether spinacetin modulates the same established target pathways.

### 3.3. Anti-Diabetic Activity of Spinacetin

Natural products are a significant reservoir of bioactive compounds with potential antidiabetic properties [36,37]. Chronic, low-grade metabolic inflammation serves as a primary driver in the pathogenesis of type 2 diabetes. Consequently, the anti-inflammatory properties of natural compounds such as spinacetin may underpin its antidiabetic potential by mitigating the inflammatory pathways. The antidiabetic potential of spinacetin, isolated from the galls of *Pistacia integerrima*, was characterized using a combination of in vitro assays and in silico molecular modeling analysis [38].

#### 3.3.1. In Vitro Antidiabetic Activity of Spinacetin

Spinacetin and five additional flavonoids isolated from the galls of *Pistacia integerrima* were evaluated for their antidiabetic potential using an α-glucosidase inhibition assay [38]. As the enzyme responsible for breaking down complex carbohydrates, α-glucosidase is a key pharmacological target for treating diabetes mellitus [39]. In the enzymatic assay, spinacetin demonstrated robust inhibition (>90%) of α-glucosidase activity, highlighting its efficacy as a potential antidiabetic agent. The findings indicate that the antidiabetic properties of the *P. integerrima* extract are largely mediated by its flavonoid constituents, specifically spinacetin and patuletin. These interactions were subsequently characterized using in silico docking studies to identify potential binding mechanisms.

#### 3.3.2. In Silico Antidiabetic Activity of Spinacetin

After validating the α-glucosidase inhibitory activity of spinacetin and related flavonoids, molecular docking was performed on a homology-modeled version of the enzyme to elucidate their binding modes [38]. To ensure the reliability of the computational parameters, the docking method was validated against acarbose and five randomly selected α-glucosidase inhibitors. Molecular docking revealed that spinacetin occupies the active site in an orientation directed toward the catalytic triad, where it forms hydrogen bonds with Asp214 and Glu276. The strong inhibition of α-glucosidase activity directly supported by the in silico docking experiments suggests the robust antidiabetic potential of spinacetin. However, diabetes is a complex disease involving several target enzymes and pathways that may also interact with spinacetin; this study prioritized its interaction with α-glucosidase. Broader polypharmacological effects were not addressed in this analysis. A major limitation of in vitro assays is their inability to simulate the multi-organ complexity of diabetes. Unlike in vivo models, they do not consider bioavailability or the intricate hormonal and neural pathways involving the liver, pancreas, and peripheral tissues. Further in vivo studies are required to establish the antidiabetic activity of spinacetin.

### 3.4. Cardioprotective Activity of Spinacetin

**Figure 1:**
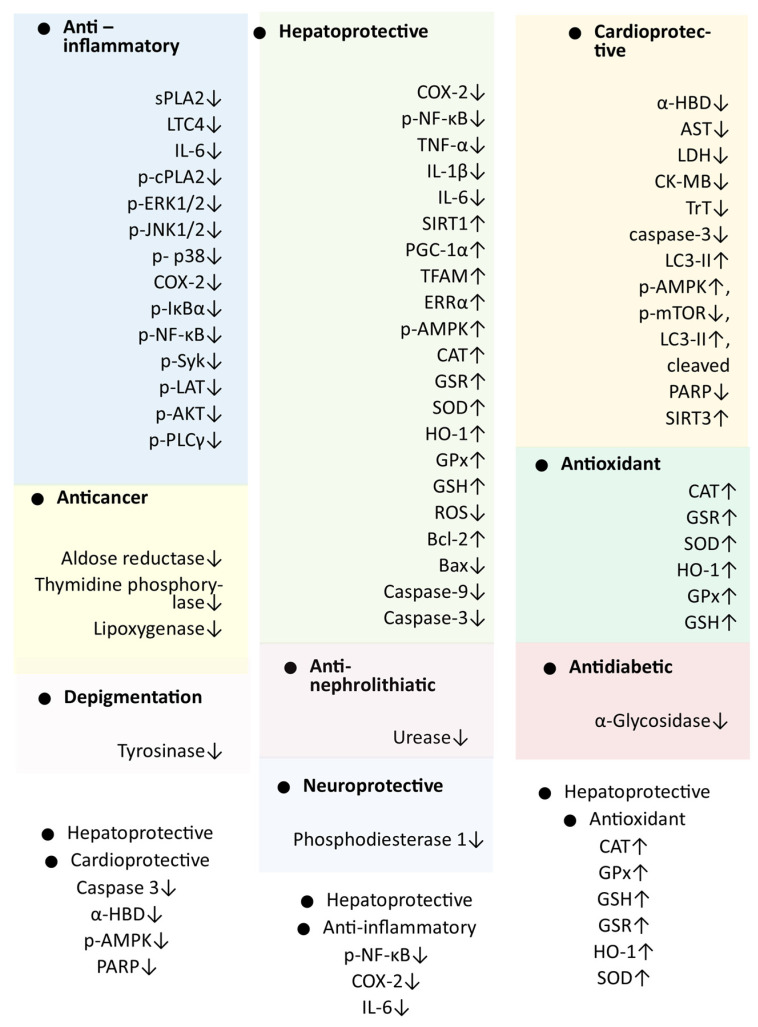
Target gene/markers associated with the pharmacological activities of spinacetin. Genes found to be associated with more than one activity are listed with a white background; ↑ denotes up-regulation, and ↓ denotes down-regulation.

Cardiovascular diseases are among the leading causes of mortality worldwide and are frequently linked to various comorbidities and pathological complications [40,41]. The diverse pharmacological activities of spinacetin have inspired researchers to evaluate its cardioprotective effects using both in vitro cell lines and animal experiments [7]. Since inflammation and oxidative stress drive both disease progression and organ toxicity, this study identified common molecular targets of spinacetin across multiple pharmacological models (Figure 1).

#### 3.4.1. In Vitro Cardioprotective Activity of Spinacetin

Doxorubicin, a chemotherapy drug known for its cardiotoxicity, was used to study the protective effects of spinacetin. The cardioprotective activity was evaluated in the H9c2 cell line, which is derived from embryonic rat heart tissue [7]. The treatment of spinacetin to H9c2 cells significantly suppressed the doxorubicin-induced cytotoxicity. To investigate the role of autophagy in the protective effect of spinacetin, the autophagy inhibitor chloroquine (CQ) was utilized. CQ administration significantly abrogated the cardioprotective effects of spinacetin, suggesting that the preservation of autophagic activity is essential for spinacetin-mediated cardioprotection. Increased apoptosis with CQ administration in spinacetin-treated cells also supports the finding. The role of spinacetin-induced autophagy in mediating its protective effects was further validated using the potent autophagy inhibitor 3-methyladenine (3-MA) and Atg7-knockdown assays via siRNA. In both cases, the spinacetin-induced upregulation of the LC3-II/LC3-I ratio (autophagy marker) and the suppression of apoptotic markers (cleaved caspase-3 and cleaved PARP) were significantly reversed (Table 3). Dansylcadaverine (MDC) staining was also employed to visualize autophagosomes, further confirming that spinacetin-induced autophagy mediates its protective effects against doxorubicin-induced cardiotoxicity. In this analysis, spinacetin treatment significantly increased autophagy as evidenced by the accumulation of MDC-stained puncta, effectively reversing the decrease caused by doxorubicin-induced toxicity. After confirmation of the role of spinacetin-induced autophagy in cardioprotection, the pathway associated with autophagy was studied. Spinacetin was found to activate AMPK by enhancing its phosphorylation, which had been suppressed by doxorubicin. Conversely, spinacetin inhibited mTOR by suppressing its phosphorylation, which was conversely upregulated by doxorubicin. Involvement of the AMPK/mTOR pathway in spinacetin-induced autophagy, the upstream regulator SIRT3, was studied. It was found that spinacetin treatment significantly increased the expression of SIRT3. To validate that SIRT3 mediated the protective effects of spinacetin against doxorubicin-induced cardiotoxicity, an siRNA-based SIRT3 knockdown was performed. In the presence of SIRT3 siRNA, spinacetin failed to activate AMPK or suppress mTOR phosphorylation. Similarly, in SIRT3-knockdown conditions, spinacetin failed to alter the LC3-II/LC3-I ratio and was unable to suppress the levels of apoptotic proteins, such as cleaved caspase-3 and cleaved PARP. Finally, flow cytometry results showed that SIRT3 knockdown abolished the ability of spinacetin to reduce the apoptotic rate following doxorubicin treatment. Collectively, these results suggest that spinacetin protects myocardial cells from doxorubicin-induced cardiotoxicity by activating the SIRT3/AMPK/mTOR signaling pathway. However, the cardioprotective activities of spinacetin have only been studied through a single model using a specific toxicity-inducing agent. Future studies are suggested to evaluate its cardioprotective activity in other models of toxicity using different toxicants such as herceptin, cyclophosphamide, or cisplatin. Furthermore, in vivo studies utilizing doxorubicin as the primary challenge agent were conducted to validate and further advance the evaluation of the cardioprotective activity of spinacetin.

#### 3.4.2. In Vivo Cardioprotective Activity of Spinacetin

In the study, 14 days of treatment with spinacetin on doxorubicin-induced cardiotoxicity was evaluated. Histopathological examination of rat heart tissue revealed that spinacetin significantly mitigated the structural damage induced by doxorubicin, preserving myocardial architecture. In the cell counting kit-8 (CCK-8) assay, spinacetin treatment alone did not induce significant cytotoxicity in primary cardiomyocytes from ICR mice; however, its administration significantly attenuated doxorubicin-induced cytotoxicity and restored the viability of these cells [7]. Additionally, common biomarkers of myocardial damage, such as lactate dehydrogenase (LDH), creatine kinase-myocardial band (CK-MB), troponin T (TrT), α-hydroxybutyrate dehydrogenase (α-HBD), and aspartate aminotransferase (AST), which were remarkably elevated in the serum by doxorubicin administration, were dose-dependently suppressed by spinacetin treatment. Myocardial malondialdehyde (MDA) level was elevated in the serum by doxorubicin administration, which was also dose-dependently suppressed by spinacetin treatment. All these results clearly showed that the spinacetin protects from doxorubicin-induced cardiotoxicity. Furthermore, consistent with the CCK-8 assay results, spinacetin suppressed doxorubicin-induced apoptosis in primary cardiomyocytes revealed by the Annexin V/PI double staining flow cytometry analysis. Cleaved caspase-3 and cleaved PARP were significantly upregulated in the doxorubicin-treated group, indicating the potent induction of apoptosis. Conversely, the treatment of spinacetin significantly attenuated the doxorubicin-induced expression of both cleaved caspase-3 and cleaved PARP.

Doxorubicin significantly attenuated the levels of the autophagy marker LC3-II, indicating the suppression of autophagic activity. Notably, the administration of spinacetin significantly upregulated LC3-II expression in primary cardiomyocytes, suggesting that spinacetin-mediated induction of autophagy may protect these cells from doxorubicin-induced apoptosis. Taken together, the findings from in vitro and animal studies demonstrate that spinacetin possesses cardioprotective effects against doxorubicin-induced cardiotoxicity. This protection is supported by the induction of autophagy, as evidenced by increased levels of the autophagy marker LC3-II in both in vitro and in vivo experiments [7]. The results suggest that spinacetin triggers autophagy via the SIRT3/AMPK/mTOR signaling cascade, which serves to protect cardiac cells against apoptotic cell death. Importantly, this study suggests that spinacetin may help mitigate cardiotoxicity, which is a major clinical concern when administering doxorubicin to cancer patients.

### 3.5. Spasmolytic Activity of Spinacetin

A spasmolytic agent, also referred to as an antispasmodic, is a pharmacologically active compound or mixture that suppresses involuntary muscle contractions [42]. These agents relieve muscle spasms and cramps by inducing smooth muscle relaxation; consequently, they are therapeutic for conditions such as gastrointestinal hypermotility, overactive bladder, and dysmenorrhea (menstrual cramps) [43].

#### In Vivo Spasmolytic Activity of Spinacetin

The potent spasmolytic activity of the extract from the aerial parts of *Artemisia copa* necessitated the identification of the specific phytoconstituents responsible for this pharmacological effect. The spasmolytic activity of spinacetin was investigated following its isolation from the extract [44]. The spasmolytic activity was studied on jejunums isolated from the female Sprague Dawley rats. In the study, spinacetin significantly suppressed the muscle contractions induced by CaCl_2_. The spinacetin exhibited non-competitive antagonism against the CaCl_2_ concentration-response curve, inhibiting maximum contractions by 49.1% upon application. The results suggest that spinacetin exerts its effect by blocking calcium influx through voltage-dependent calcium channels. The study suggests that spinacetin, along with other phytochemicals possessing spasmolytic properties, is a major constituent responsible for the spasmolytic activity of the extract [44]. However, spasmolytic activity has only been studied in a single experimental model. In vivo models, such as the charcoal meal transit test and castor-oil-induced diarrhea studies, are strongly suggested to validate these activities [45].

### 3.6. Analgesic, Sedative and Muscle Relaxant Activities of Spinacetin

#### 3.6.1. In Vivo of Analgesic, Sedative and Muscle Relaxant Activities of Spinacetin

The pharmacological profile of spinacetin, including its sedative and muscle relaxant activities, was examined after its isolation from the methanolic extract of *Euphorbia pulcherrima*. Four doses (5, 10, 15, and 20 mg/kg) of spinacetin were analyzed to study its pharmacological activities in mouse models. The sedative potential of spinacetin was evidenced by a significant dose-dependent reduction in locomotor activity, as observed by the decreased frequency of line crossings in a custom-designed activity box. Similarly, the muscle relaxant potential of spinacetin was evaluated using the inclined plane and traction tests, both of which revealed significant muscle relaxant activity. In both experiments, the effects of spinacetin were evaluated at 30, 60, and 90 min post-administration, revealing both dose- and time-dependent activities. Like sedative and muscle relaxant activities, the analgesic activities of the flavonoids spinacetin, isolated from the methanolic extract of *Euphorbia pulcherrima*, were evaluated in Balb/c mice.

Analgesic activity was evaluated at 30, 60, 90, and 120 min post-administration for four doses of spinacetin (5, 10, 15, and 20 mg/kg) using a hot-plate analgesiometer. Spinacetin exhibited significant and sustained dose-dependent analgesic effects for up to 120 min following administration.

The study suggested that the active flavonoids, such as spinacetin and patuletin, are the major components responsible for the activities of *Euphorbia pulcherrima* used in the folk medicine [11].

#### 3.6.2. In Silico Analgesic Activity of Spinacetin

Following the in vivo demonstration of its analgesic activity, a molecular docking study was conducted on pain-related proteins to investigate the potential interactions and underlying mechanisms of spinacetin. Molecular docking of spinacetin was conducted on GABA_A_ and μ-opioid receptors after validating the docking protocols with standard reference ligands.

γ-Aminobutyric acid type A (GABA_A_) and *μ*-opioid receptor were used for the docking simulation for docking of spinacetin after docking parameter optimization on known ligands. In the docking study, spinacetin exhibited effective binding interactions with the GABA_A_ receptor. The binding energy and *Ki* for this complex were −6.49 kcal/mol and 17.44 μM, respectively. Similarly, strong interactions were observed with the *μ*-opioid receptor, spinacetin showed a higher affinity, with a binding energy of −7.89 kcal/mol and a *Ki* value of 4.84 μM. Results from the molecular studies support the in vivo analgesic activity of spinacetin through GABA_A_ and *μ*-opioid receptors. However, the potential involvement of GABA_A_ and μ-opioid receptors remains unverified through lab experiments, and several other targets are also known for analgesic activity; thus, in vitro studies are required to validate spinacetin’s molecular targets.

### 3.7. Antioxidant Activity of Spinacetin

Antioxidant-rich foods are preferred due to their numerous health benefits and their role in managing various diseases and physiological conditions [46]. Due to concerns regarding the toxicity and side effects of synthetic antioxidants used as food additives, natural alternatives are increasingly favored for their safety and health benefits [47]. In the search for bioactive constituents, the antioxidant activity of spinacetin was evaluated following its isolation from different medicinal plants.

#### In Vitro Antioxidant Activity of Spinacetin

Pharmacological studies of *Inula britannica* support its traditional use in treating gastrointestinal ailments, respiratory infections such as bronchitis, and various inflammatory or neoplastic diseases [6,26,48]. Phytochemical characterization and antioxidant assays were performed on the isolated compounds to identify the primary active constituents of the plant [48]. The antioxidant effect of all isolated compounds, including spinacetin (one of the major compounds), was evaluated by measuring their 2,2-Diphenyl-1-picrylhydrazyl (DPPH) radical scavenging. The spinacetin was found to be highly active in the scavenging of DPPH free radical (85.4%) in the study. These findings suggest that highly active compounds, particularly spinacetin, are likely the primary contributors to the overall biological activity of the plant.

Later, *Chrysanthemum morifolium* flowers, which are widely used in traditional Chinese medicine and known for their high antioxidant content, were used to isolate several flavonoids [5]. The antioxidant and anti-inflammatory activities of these isolated compounds were subsequently evaluated. The antioxidant and anti-inflammatory effects of all isolated compounds, including spinacetin, were evaluated by measuring their DPPH radical-scavenging activity and their inhibitory effects on NO production in LPS-induced RAW 264.7 cells (Table 4). In the study, spinacetin demonstrated potent DPPH radical-scavenging activity and significant inhibition of NO production [49]. Notably, the IC_50_ value (17.22 ± 1.23 μM) for spinacetin-mediated inhibition of NO production was superior to that of the positive control, ibuprofen (IC_50_ = 30.57 μM). The structure–activity relationship suggested that the 3′,4′-dimethoxylations in the B-ring tended to enhance the activities. Spinacetin showed strong antioxidant potential in radical scavenging assays, outperforming positive controls in cellular models, which aligns with the antioxidant properties of the flavonoid class [50,51]. The in vivo antioxidant effects of spinacetin on various antioxidant enzymes are detailed in the hepatoprotective activity section.

### 3.8. Miscellaneous Activities of Spinacetin

The pharmacological profiles of spinacetin and patuletin, which were isolated from *Euphorbia pulcherrima* bark, were assessed through integrated in vitro and in silico methodologies. In the study, spinacetin was assessed for its inhibitory activity against three clinically relevant enzymes: urease, tyrosinase, and phosphodiesterase 1 [14]. Urease serves as a primary clinical target for managing urinary tract infections and preventing the crystallization of kidney stones induced by ureolytic bacteria [14]. Similarly, tyrosinase is the central target in hyperpigmentation disorders, frequently utilized in the cosmetic industry as a skin-whitening agent [13]. The phosphodiesterase inhibition plays a critical role in treating various pathologies, including cardiovascular and neurological diseases [12,52]. Spinacetin demonstrated potent phosphodiesterase 1 (PDE1) inhibitory activity, suggesting its potential role in neuroprotection and cardioprotection. However, further studies are required to validate these pharmacological effects. The urease inhibitory activity of spinacetin was found to be superior to that of the positive control, thiourea, suggesting that spinacetin has significant potential as a potent urease inhibitor. Similarly, spinacetin exhibited tyrosinase inhibition levels similar to those of the positive control, kojic acid, demonstrating its effectiveness as a tyrosinase inhibitor (Table 5).

#### In Silico Miscellaneous Activities of Spinacetin

Based on the effective in vitro inhibition of urease, tyrosinase, and phosphodiesterase 1 by spinacetin, in silico docking simulations were carried out for all three enzymes [14]. The crystal structures of urease and tyrosinase were retrieved from the PDB, while the structure of phosphodiesterase 1 was generated via homology modeling for the docking studies. Molecular docking analysis using AutoDock Vina showed that spinacetin outperformed the standard positive controls across all three target enzymes, yielding more favorable docking scores. Interaction analysis of the spinacetin and urease complex revealed a hydrogen bond between a hydroxyl group of spinacetin and His541, alongside six hydrophobic interactions. Similarly, the docking complex of spinacetin and tyrosinase exhibited six hydrophobic interactions. Spinacetin exhibited robust binding with phosphodiesterase-1, forming three hydrogen bonds and nine hydrophobic interactions. The docking results support the in vitro inhibitory activities of spinacetin against these enzymes. However, it should be noted that the docking protocol was not validated using a re-docked co-crystal ligand (or known ligand) prior to the actual docking studies.

### 3.9. Antileishmanial Activity of Spinacetin

Leishmaniasis, caused by parasites of the genus *Leishmania*, is the third most significant vector-borne parasitic disease globally and can be fatal if left untreated [54]. Current leishmaniasis treatments are limited by high costs and significant toxicity, driving the need for better alternatives. Natural products offer a promising reservoir for drug discovery due to their diverse biological activities and relatively fewer side effects [54,55].

#### 3.9.1. In Vitro Antileishmanial Activity

Due to the traditional use of *Pistacia chinensis* in treating leishmaniasis, spinacetin and other compounds were isolated from the plant and evaluated for their antileishmanial activity. The in vitro antileishmanial activity was evaluated against *Leishmania major* cultures, using a standard drug (amphotericin B) as a positive control. Efficacy was determined by quantifying live and dead promastigotes via light microscopy. The significant antileishmanial activity of spinacetin (IC_50_ = 9.23 ± 0.23 μM), which was the second highest among the tested compounds after sakuranetin (IC_50_ = 7.98 ± 0.16 μM), was observed in the study. Effective in vitro antileishmanial activity inspired researchers to decipher the molecular target of spinacetin in further in silico studies.

#### 3.9.2. In Silico Antileishmanial Activity

In silico molecular docking studies were performed against the established *Leishmania major* drug targets, dihydrofolate reductase (DHFR) and pteridine reductase 1 (PTR1). The crystal structure of DHFR was retrieved from the PDB, while a homology-modeled structure was utilized for PTR1 in the molecular docking studies. In line with in vitro results, spinacetin (−6.32 kcal/mol) was the second highest among the tested compounds after sakuranetin (−6.48 kcal/mol) in the DHFR docking study. In the case of PTR1, spinacetin showed a higher docking score compared to the sakuranetin. Interaction analysis of docked complexes also showed several interactions between spinacetin and target proteins. Overall, spinacetin exhibited significant in vitro antileishmanial effects, with in silico results suggesting DHFR and PTR1 as likely targets. However, several other antileishmanial targets have been reported [36]; future studies involving experimental validation are necessary to confirm these specific molecular interactions.

### 3.10. Hepatoprotective Activity of Spinacetin

Hepatic disorders cause a significant burden on public health, and natural compounds may be potential candidates for hepatoprotective activity [56,57]. Similarly to the cardioprotective activity of spinacetin, its hepatoprotective activity and the underlying molecular mechanisms were studied through a series of relevant animal and molecular experiments.

#### In Vivo Hepatoprotective Activity

The hepatoprotective activity of spinacetin was studied in cadmium (Cd)-exacerbated hepatic ischemia/reperfusion (I/R) injury in rats. In the study, a 50 mg/kg dose of spinacetin was evaluated against I/R injury in rats. The liver damage markers such as AST, gamma-glutamyl transferase (GGT), alanine transaminase (ALT), and alkaline phosphatase (ALP) were upregulated due to I/R injury in the rats, which were further enhanced with Cd toxicity [9]. However, the levels of albumin and total protein were suppressed due to injury. Conversely, spinacetin treatment suppressed all these liver damage markers and enhanced the levels of albumin and total protein. Histopathological analysis revealed that the I/R injury group exhibited significant hepatic damage, characterized by hepatocytic degeneration, vascular congestion, irregular hepatocyte size, and mild fibrosis compared to the control. However, the combination of Cd administration and I/R injury resulted in severe architectural disruption. This group displayed extensive necrosis, hemorrhage, vacuolar degeneration, and severe inflammation compared to the I/R-only group. Notably, spinacetin treatment led to significant histological recovery in both conditions (I/R-only and Cd administration + I/R injury) compared to their respective non-treated counterparts. Consistent with the histopathology results, the pro-apoptotic markers (Bax, Caspase-9, and Caspase-3) were increased, while the anti-apoptotic marker (Bcl-2) was suppressed in the I/R group compared with the normal group. The I/R + Cd group exhibited a similar trend in the expression of these markers to the I/R group, though the observed effects were significantly more prominent. However, the spinacetin treatment significantly reversed the expression of these markers in both conditions (I/R-only and Cd administration + I/R injury) compared to their respective non-treated counterparts.

The gene expression of AMPK, SIRT1, PGC-1α, TFAM, and ERRα was found to be significantly suppressed due to I/R injury, which was further aggravated by the administration of Cd (Cd + I/R injury). Spinacetin administration significantly upregulated the expression of these genes, effectively mitigating the suppressive effects of both I/R injury and Cd + I/R injury. Conversely, the expression of NF-κB, IL-6, IL-1β, TNF-α, and COX-2 was significantly suppressed by the spinacetin, which was enhanced due to I/R injury and Cd + I/R injury.

The effects of spinacetin on activities of antioxidant enzymes such as CAT, glutathione reductase (GSR), SOD, heme-oxygenase-1 (HO-1), GPx, levels of glutathione (GSH), reactive oxygen species (ROS), and MDA were assessed in rats subjected to I/R and Cd + I/R injury. The oxidative stress markers ROS and MDA were found to be increased due to I/R injury, which was further enhanced by the administration of Cd (Cd + I/R injury). However, the spinacetin treatment suppressed both of these markers in the study. Conversely, I/R injury reduced GSH (antioxidant) levels and suppressed the activities of SOD, CAT, GPx, HO-1, and GSR (antioxidant enzymes). These reductions were further aggravated in the Cd + I/R injury group. Spinacetin administration significantly restored the level of antioxidants and activities of antioxidant enzymes (Table 6). Spinacetin not only suppressed liver damage marker enzymes but also alleviated histopathological damage, as observed in the tissue staining. This protection was further supported by the suppression of pro-apoptotic markers and the enhancement of anti-apoptotic protein levels. Importantly, the hepatoprotective activity of spinacetin can be compared to that of similar phytochemicals, such as quercetin, kaempferol, and luteolin. These compounds have also demonstrated hepatoprotective effects through the suppression of inflammatory NF-κB signaling pathways and ROS production, alongside the activation of AMPK signaling pathways [58,59,60]. However, while the hepatoprotective activities of these phytochemicals have been extensively evaluated across multiple animal models, spinacetin has thus far been studied in only a single model.

## 4. Limitations

The therapeutic development of spinacetin remains hindered by both activity-specific gaps and general pharmacological limitations (Table 6). One of the significant limitations in the current pharmacological development of spinacetin is the lack of extensive safety studies. However, in a toxicological study, the intraperitoneal administration of spinacetin at doses up to 200 mg/kg was found to be safe in the Balb/c mice used in the study [11]. Considering the broad pharmacological potential of spinacetin, longer-term safety assessments, including chronic toxicity studies and detailed organ-specific evaluations, are essential. Furthermore, since different routes of administration can result in distinct toxicity profiles, future research should investigate the safety of spinacetin via various delivery methods such as oral, intravenous, and topical applications. Robust pharmacokinetic studies for spinacetin are currently missing from the literature. Pharmacokinetic studies can provide insights into the bioavailability of spinacetin within various organs, such as the liver and heart. Furthermore, understanding its tissue distribution may help optimize the protective role of spinacetin in these vital organs. However, a study not primarily focused on spinacetin revealed its detection in plasma following the bolus ingestion of spinach. Comprehensive pharmacokinetic studies on spinacetin are essential for the rational design of dosing regimens in future animal and clinical studies. Importantly, the unavailability of animal studies restricts the therapeutic evaluation of spinacetin against complex metabolic disorders, such as diabetes and cancer.

Several in silico docking studies have also been conducted to evaluate the therapeutic potential of spinacetin against key drug targets in different diseases. However, caution is required during the assessment of findings from docking studies. Molecular docking results that are not validated using known ligands and/or compared with experimental data may lack accuracy and reliability. For example, the docking of spinacetin onto tubulin lacks practical significance without experimental validation, as computational predictions must be confirmed through biochemical or cellular assays [10].

## 5. Conclusions and Future Directions

Spinacetin has shown multiple pharmacological activities in various studies. While some are in the preliminary stages of development; others are more significant. The anti-inflammatory and antioxidant activities of spinacetin are apparent through numerous in vitro and animal studies [9,11,24,49]. These activities are considered crucial as they contribute to the prevention and management of serious diseases, including cancer and diabetes, while offering protection to vital organs such as the heart and liver [7,9].

The anti-inflammatory activity of spinacetin was demonstrated in three animal models, specifically PCA and both carrageenan-induced and histamine-induced paw edema, suggesting a robust therapeutic potential. Further, the suppression of inflammatory enzymes like sPLA2 (linked to arthritis), COX-2, and iNOS provides a mechanistic basis for inflammation control. This was supported by the downregulation of key signaling pathways such as PI3K/AKT, MAPK, and NF-κB, alongside upstream molecules LAT and Syk. Given the strong anti-inflammatory basis of spinacetin, there is a clear rationale to expand its protective activity to other organs, such as the lungs, spleen, colon, and brain. Notably, its ability to suppress PDE1 also supports its potential neuroprotective activity [14].

ROS and other oxidants are produced during metabolic processes. Excessive production of these molecules can disrupt cellular homeostasis and is associated with numerous diseases; much like inflammation, this oxidative stress can lead to systemic toxicity in various organs. The antioxidant activity of spinacetin, which was higher than that of the positive control in radical scavenging assays, is also evidenced by the enhancement of crucial antioxidant enzymes (CAT, GSR, SOD, HO-1, and GPx) in the liver [9]. Both of these, the enzymatic and non-enzymatic antioxidant activities of spinacetin, may contribute significantly to its hepatoprotective effects. This is evidenced by the enhancement of antioxidant enzymes and antioxidant levels in the liver, which were previously suppressed by Cd toxicity and ischemia–reperfusion injury [9]. This activity likely contributes to its broader pharmacological properties and the protection of vital organs against oxidative stress. For example, the level of MDA, a key marker of lipid peroxidation and oxidative stress, was significantly suppressed by spinacetin in the case of doxorubicin-induced cardiotoxicity [7]. Similarly to its anti-inflammatory properties, the antioxidant activity of spinacetin contributes to a broad pharmacological profile, warranting further investigation into its protective effects on various organs in future studies.

The anticancer activity of spinacetin against various cancer types, combined with its non-toxic effects on normal cells, highlights its therapeutic potential [8,10]. Notably, spinacetin outperformed the positive control (7-deazaxanthine) in the enzyme inhibition assay [32]. While the specific anticancer mechanisms of spinacetin remain largely uncharacterized, its potential may lie in the modulation of pro-inflammatory pathways associated with malignancy. Given that spinacetin targets the NF-κB and MAPK signaling axes in inflammatory cells (Table 1), these pathways warrant further investigation to fully elucidate its anticancer potential. However, the lack of in vivo studies in complex diseases, such as diabetes and cancer, restricts the current status of spinacetin as a candidate for anticancer and antidiabetic development. Therefore, validation of both activities in animal studies is strongly suggested in future research. Similarly, spasmolytic activity is suggested to be validated through other animal studies. Additionally, identifying its molecular targets would be essential for optimizing spasmolytic activity during development. Animal studies demonstrated that spinacetin possesses muscle relaxant, sedative, and analgesic properties. The overlap of these effects suggests that spinacetin may interact with a common molecular target responsible for modulating these related functions. Molecular docking studies supported the roles of GABA_A_ and *μ*-opioid receptors in analgesic activities. However, because multiple targets are associated with analgesic effects, experimental validation of these receptor interactions is strongly suggested. The inhibition of COX-2 observed in the anti-inflammatory assays may also contribute to the spinacetin’s analgesic effects [24,61].

Spinacetin not only suppressed liver damage marker enzymes (ALT, AST, ALP, and GGT) but also alleviated histopathological signs of damage. These findings were further supported by the suppression of pro-apoptotic markers (Bax, Caspase-9, and Caspase-3) and the enhancement of anti-apoptotic marker proteins (Bcl-2). Notably, enhancement of antioxidant enzymes, suppression of oxidants markers, were observed with spinacetin treatment. Similarly, the inflammatory markers and pathways were also suppressed by the spinacetin in Cd toxicity and ischemia–reperfusion injury. These findings clearly demonstrated the role of antioxidant and anti-inflammatory activity of spinacetin in hepatoprotection. This robust hepatoprotective mechanism has the potential to protect the liver against a diverse range of stressors, including ethanol, high-fat diets, carbon tetrachloride-induced toxicity, and fatty liver disease. While the investigation was focused on ischemia–reperfusion injury, future studies are warranted to evaluate the efficacy of spinacetin in these additional models.

The cardioprotective activity of spinacetin was evident through both in vitro and in vivo experiments. Similarly to its hepatoprotective effects, spinacetin’s antioxidant role was demonstrated through the suppression of oxidative markers such as malondialdehyde, thereby contributing to its cardioprotective effects. The induction of autophagy was found to be a key mechanism underlying spinacetin’s cardioprotective activity, as validated through various experimental assays, including the administration of autophagy inhibitors (CQ and 3-methyladenine), Atg5 knockdown assays, and MDC staining to visualize autophagosomes. Autophagy is a critical, lysosome-mediated ‘self-cleaning’ process that maintains cellular homeostasis by degrading damaged organelles and misfolded proteins; its induction may alleviate various conditions, including neurodegenerative disorders and various types of cancer. Given the strong evidence of spinacetin-induced autophagy, its pharmacological applications could be expanded to neurodegenerative disorders such as Alzheimer’s disease (AD). Furthermore, additional studies should be conducted to explore the role of autophagy in the anticancer activity of spinacetin.

Spinacetin was found to target several crucial molecular targets and signaling pathways, highlighting its significant pharmacological potential against various diseases (Figure 1). Those targets (such as targets associated with inflammation) and pathways (such as AMPK and NF-κB) identified as common across different pharmacological activities may be considered more reliable and should be prioritized in future studies (Figure 1).

Other significant activities, including neuroprotective, anti-nephrolithiatic, and depigmentation effects, also show potential for development. However, these activities are currently supported only by the inhibition of respective target enzymes in in vitro studies, further supported by molecular docking simulations [14]. Because the roles of pharmacokinetics and bioavailability are missing in these studies, animal studies are essential for confirming therapeutic potential and optimizing the dosage of spinacetin for subsequent studies.

The significant pharmacological activities of spinacetin, such as its anti-inflammatory, anticancer, and hepatoprotective effects, place it within the category of potent bioactive compounds like kaempferol, quercetin, and luteolin. However, while these reference compounds have been extensively studied for their mechanisms of action and validated through various animal models and clinical trials, such comprehensive data are currently lacking for spinacetin. Importantly, these compounds also positively modulate the gut microbiome, which has been identified as a contributing factor to their pharmacological activities [29,58,60]. However, the effect of spinacetin on the gut microbiota has not yet been investigated; thus, exploring this relationship in future studies is essential to fully understanding its pharmacological profile.

Overall, spinacetin is a promising phytochemical that can be subjected to animal and advanced studies as per the suggestions. As most research on spinacetin is currently in the preclinical stage (in vitro and in vivo), and comprehensive pharmacokinetic and safety profiles are lacking, small-scale Phase I clinical trials focusing on safety should only be conducted following successful pharmacological validation and rigorous toxicity evaluations.

## Figures and Tables

**Table 1:** Anti-inflammatory activities of spinacetin.

Type of Study	Compound Source and Dose	Method	Standard/Control Result	Ref.
In vitro	Spinacetin isolated from *Artemisia copa* at 10 μM	LPS-stimulated RAW 264.7 macrophages were used for Radioimmunoassay for PGE_2_	Production of PGE_2_↓	[24]
		Nitrile level was determined by fluorometric method	Production of NO↓,	
		sPLA_2_ activity by using [^3^H]oleate labeled membrane of *Escherchia coli*	sPLA_2_↓	
	Spinacetin isolated from *Inula japonica* at 1, 2, and 5 μM	Intracellular Ca^2+^ level was determined with the FluoForte Calcium assay in IgE/Ag-stimulated BMMCs	Intracellular Ca^2+^↓	[25]
		Immunoassay IgE/Ag-stimulated BMMCs	Histamine degranulation↓, LTC4↓ and IL-6↓	
		WB of IgE/Ag-stimulated BMMCs	phosphorylation and nuclear translocation of cPLA2↓, phosphorylation of ERK1/2, JNK1/2, and p38↓, IL-6 cytokine levels↓ COX-2 expression↓, phosphorylation and subsequent degradation of IκBα↓, NF-κB translocated from the cytosol to the nucleus↓ and phosphorylation of AKT↓	
		Immunoprecipitation	Phosphorylation of both Syk and LAT↓	
		WB of IgE/Ag-stimulated RBL-2H3 cells	Phosphorylation and nuclear translocation of cPLA_2_↓, Phosphorylation of ERK1/2, JNK1/2, and p38↓, phosphorylation and subsequent degradation of IκBα↓, NF-κB translocated from the cytosol to the nucleus, phosphorylation of AKT↓, and phosphorylation of PLCγ↓	
In vivo	Spinacetin isolated from *Inula japonica* at 25, and 50 mg/kg orally administered 1 h prior to DNP-HSA.	ICR mice were used for PCA model (intravenously challenged with 60 mg of DNP-HSA) and dexamethasone is used as positive control	PCA reaction↓ (amount of diffused dye↓ and ear thickness↓)	[25]
	Spinacetin isolated from *Euphorbia pulcherrima* at 5, 10, 15, and 20 mg/kg i.p. 30 min prior to challenge	Balb/c mice carrageenan-induced paw edema models diclofenac	Paw edema↓	[11]
		Balb/c mice histamine-induced paw edema models in Balb/c mice. (loratadine)	Paw edema↓	

AKT: protein kinase B; BMMCs: bone marrow-derived mast cells; COX-2: cyclooxygenase-2; cPLA_2_: cytosolic phospholipase A_2_; DNP-HSA: 2,4-Dinitrophenylated Human Serum Albumin; ERK: extracellular signal-regulated kinases; i.p.: intraperitoneal; JNK: c-Jun N-terminal kinases; LTC4: leukotrienes C4; PLCγ: NO: nitric oxide; IgE: immunoglobulin E; Ag: antigen; PGE_2_: Prostaglandin E2; sPLA_2_: human synovial lipase A_2_; LPS: lipopolysaccharide; Syk: spleen tyrosine kinase; LAT: linker for activation of T cells; PCA: passive cutaneous anaphylaxis; IL: interleukin; NF-κB: nuclear factor kappa-light-chain-enhancer of activated B cells; IκBα: nuclear factor kappa-light-chain-enhancer of activated B cells inhibitor-α; WB: Western blot.

**Table 2:** Anticancer activities of spinacetin.

Type of Study	Compound Source and Dose	Method	Standard/Control Result	Ref.
In vitro	Spinacetin isolated from *Pistacia integerrima*	MTT assay against HepG2, A498, NCI-H226, and MDR2780 AD cell lines	IC_50_ values for HepG2, A498, NCI-H226, and MDR2780 AD cell lines were 20.8 ± 0.33, 118.54 ± 0.023, 72.87 0.21, and 0.60 0.32 μM, respectively.	[10]
	Spinacetin isolated from *Pistacia chinensis*	Aldose reductase inhibition assay	IC_50_ = 8.2 ± 0.2 μM	[32]
		Thymidine phosphorylase inhibition assay	IC_50_ = 1.9 ± 0.9 μM	
	Spinacetin isolated from *Pistacia integerrima*	Lipoxygenase inhibitory assay	IC_50_ = 40.34 ± 1.03 μM	[8]

MTT: 3-(4,5-dimethylthiazole-2-yl)-2,5-diphenyl tetrazolium bromide; IC_50_: half maximal inhibitory concentration.

**Table 3:** Cardioprotective activities of spinacetin.

Type of Study	Compound Source and Dose	Method	Standard/Control Result	Ref.
In vitro	Spinacetin at 10 μM	H9c2 cell line doxorubicin-induced cytotoxicity (4.235 μM.)	Cytotoxicity↓	[7]
		Annexin V-PI double staining method	Apoptosis↓	
		Dansylcadaverine (MDC) staining	MDC-stained puncta↑	
		WB	Phosphorylation of AMPK↑, phosphorylation of mTOR↓, LC3-II/LC3-I ratio↑, cleaved caspase-3↓, cleaved PARP↓, and SIRT3↑	
In vivo	Spinacetin at 50 and 100 mg/kg doses intragastrically for 14 days	Histopathological examination in ICR mice	Heart tissue damage↓	[7]
		Viability study on primary cardiomyocytes from ICR mice in CCK-8 assay	Cytotoxicity↓	
		Primary cardiomyocytes Annexin V/PI double staining and flow cytometry analysis	Apoptosis↓	
		Serum ELISA and other assays	Serum level of α-HBD↓, AST↓, LDH↓, CK-MB↓, and TrT↓	
		Lipid peroxidation assay of heart tissue	MDA↓	
		WB	Cleaved caspase-3↓ and cleaved PARP↓, LC3-II expression↑ in primary cardiomyocytes	

AMPK: 5′ adenosine mono-phosphate-activated protein kinase; AST: aspartate aminotransferase; ELISA: enzyme-linked immunosorbent assay; CCK-8: cell counting kit-8; LDH: lactate dehydrogenase; CK-MB: creatine kinase-myocardial band; TrT: troponin T; α-HBD: α-hydroxybutyrate dehydrogenase; MDA: malondialdehyde; WB: Western blot; PARP: poly(adenosine diphosphate-ribose) polymerase; LC-3: microtubule-associated protein 1 light chain 3; SIRT: sirtuin; PI: propidium iodide.

**Table 4:** Antioxidant activities of spinacetin.

Type of Study	Compound Source and Dose	Method	Standard/Control Result	Ref.
In vitro	Spinacetin isolated from *Inula Britannica* at 20–100 μM	DPPH radical-scavenging activity	85.4% radical-scavenging activity by spinacetin at 100 μM	[48]
	Spinacetin isolated from *Chrysanthemum morifolium* at various doses	DPPH radical-scavenging activity	SC_50_ = 67.05 ± 5.48 μM	[49]
		NO production in LPS-induced RAW 264.7 cells	IC_50_ = 17.22 ± 1.23 μM	

DPPH: 2,2-diphenyl-1-picrylhydrazyl; LPS: lipopolysaccharide; IC_50_: half of maximal inhibitory concentration; SC_50_: represents the concentration of a substance required to scavenge 50% of free radicals.

**Table 5:** Other pharmacological activities of spinacetin.

Type of Study	Activity	Compound Source and Dose	Method	Standard/Control Result	Ref.
In vitro	Anti-nephrolithiatic	Spinacetin isolated from *Euphorbia pulcherrima* bark.	Urease inhibition assay	IC_50_ = 15.3 ± 2.13 μM	[14]
	Depigmentation	Spinacetin isolated from *Euphorbia pulcherrima* bark.	Tyrosinase inhibition assay	IC_50_ = 48.7 ± 2.19 μM	
	Antidiabetic activity	Spinacetin isolated from the galls *Pistacia integerrima* at 0.2 μg.	In Vitro α-Glycosidase Assay	IC_50_ = 826.43 ± 1.87 μM	[38]
	Neuroprotective activity	Spinacetin isolated from *Euphorbia pulcherrima* bark.	Phosphodiesterase 1 inhibition assay	IC_50_ = 148.7 ± 1.09 μM	[14]
	Antileishmanial activity	Spinacetin isolated from the *Pistacia chinensis*.	Culture of *Leishmania major* was used for quantifying live and dead promastigotes via light microscopy.	IC_50_ = 9.23 ± 0.23 μM	[53]
In vivo	Analgesic	Spinacetin isolated from *Euphorbia pulcherrima* at 5, 10, 15, and 20 mg/kg orally.	Balb/c mice were studied by hot-plate analgesiometer (30, 60, 90, and 120 min).	Time of thermal tolerance↑	[11]
	Hepatoprotecive	Spinacetin 50 mg/kg doses orally for 10 days.	Male albino rats ischemic reperfusion procedure and ischemic reperfusion procedure + Cd toxicity. Histopathological assessment (H & E staining).	hepatic damage↓	[9]
			qRT-PCR	COX-2↓, NF-κB↓, TNF-α↓, IL-1β↓, IL-6↓, SIRT1↑, PGC-1α↑, TFAM↑, ERRα↑, and AMPK↑	
			Antioxidants enzymes inhibition assays.	CAT↑, GSR↑, SOD↑, HO-1↑, and GPx↑	
			Level of antioxidant markers.	GSH↑, ROS↓	
			ELISA	AST↓, GGT↓, ALT↓, ALP↓, Bcl-2↑, Bax↓, Caspase-9↓, and Caspase-3↓	
	Sedative	Spinacetin isolated from *Euphorbia pulcherrima* at 5, 10, 15, and 20 mg/kg i.p.	Balb/c mice were used for sedative activity (frequency of line crossings in a custom-designed activity box after 30 min of administration).	No. of line cross↓	[11]
	Muscle relaxant	Spinacetin isolated from *Euphorbia pulcherrima* at 5, 10, 15, and 20 mg/kg i.p. in Balb/c mice.	Inclined plane test (after 30, 60, and 90 min of administration).	Time of thermal tolerance↑	
			Traction test(after 30, 60, and 90 min of administration).	Time of thermal tolerance↑	
	Spasmolytic activity	Spinacetin isolated from *Artemisia copa* at 15 and 30 μg mL^−1^ 30 min before study.	Female Sprague Dawley rats. Jejunums the muscle contractions induced by CaCl_2_.	Maximum contractions↓ by 49.1%	[44]

AMPK: 5′ adenosine mono-phosphate-activated protein kinase; AST: aspartate aminotransferase; COX-2: cyclooxygenase-2; GGT: gamma-glutamyl transferase; ALT: alanine transaminase; ALP: alkaline phosphatase; ELISA: enzyme-linked immunosorbent assay; IL: interleukin; i.p.: intraperitoneal; NF-κB: nuclear factor kappa-light-chain-enhancer of activated B cells; PGC-1α: peroxisome proliferator-activated receptor-γ coactivator 1-α; TFAM: Transcription factor A mitochondrial; ERRα: Estrogen-related receptor α; TNF-α: tumor necrosis factor α; Bcl-2: B-cell lymphoma 2; Bax: B-cell lymphoma 2 associated X protein; CAT: catalase; GSR: glutathione reductase; SOD: superoxide dismutase; HO-1: heme-oxygenase-1; and GPx: glutathione Peroxidase.

**Table 6:** Summary of significant pharmacological activities, highlighting core findings, limitations, and shared mechanisms of action.

Activity	Evidence Level	Core Findings	Shared Signaling Axes/Activities	Gaps/Limitations	Ref.
Anti-inflammatory activity	In silico, in vitro, and in vivo studies	Targeting important inflammatory pathways and suppression of inflammation in different types of cells and animal models	p-NF-κB↓, COX-2↓, IL-6↓/Observed in hepato and cardioprotective activities	Limited study on inflammatory-associated disease models and clinical studies.	[11,24,49]
Antioxidant activities	In vitro assays, and in vivo studies	Enzymatic and non-enzymatic antioxidant activities were observed	MDA, antioxidant enzymes/Observed in hepato and cardioprotective activities	Limited study on antioxidant-associated disease models and clinical studies.	[48,49]
Cardioprotective	In vitro cell line and in vivo studies	Protective activity was observed in cellular and histopathological studies, highlighting the modulation of key molecular targets.	Caspase 3↓, α-HBD↓, p-AMPK↓, PARP↓/anti-oxidant and hepatoactivities	Only single animal models have been used till now to reveal cardioprotective activity.	[7]
Hepatoprotective activity	In silico and in vivo studies	Protective activity was observed in histopathological experiments, highlighting the modulation of key targets.	Caspase 3↓, α-HBD↓, p-AMPK↓, PARP↓/anti-oxidant and cardioprotective activities	Only single animal models have been used till now to reveal hepatoprotective activity.	[9]
Anticancer	In silico and in vitro studies	Effective against multiple types of cancer cells and provides inhibition of key target enzymes.	Anti-inflammatory pathways signaling and activity may be contributing to anticancer activity (not studied in anticancer activity).	Limited mechanistic studies and missing in vivo experiments.	[8,10,32]
Analgesic	In silico and in vivo studies	Effective analgesic activity in animal model and suppression of target enzyme.	COX-2/anti-inflammatory activity	Only single models have been used till now; more in vivo models are required to validate the activity.	[11]

AMPK: 5′ adenosine mono-phosphate-activated protein kinase; α-HBD: α-hydroxybutyrate dehydrogenase; MDA: malondialdehyde; PARP: poly(adenosine diphosphate-ribose) polymerase; COX-2: cyclooxygenase-2; IL: interleukin; NF-κB: nuclear factor kappa-light-chain-enhancer of activated B cells.

## Data Availability

No new data were created or analyzed in this study. Data sharing is not applicable to this article.
